# Bandgap and refractive index estimates of InAlN and related nitrides across their full composition ranges

**DOI:** 10.1038/s41598-020-73160-7

**Published:** 2020-10-01

**Authors:** Shahab N. Alam, Vitaly Z. Zubialevich, Bijan Ghafary, Peter J. Parbrook

**Affiliations:** 1 Tyndall National Institute, Lee Maltings Dyke Parade, Cork, Ireland; 2 School of Engineering, University College Cork, Cork, Ireland; 3 Physics Department, Iran University of Science and Technology, 16846 Tehran, Iran

**Keywords:** Materials for optics, Lasers, LEDs and light sources

## Abstract

III-Nitride bandgap and refractive index data are of direct relevance for the design of (In, Ga, Al)N-based photonic and electronic devices. The bandgaps and bandgap bowing parameters of III-nitrides across the full composition range are reviewed with a special emphasis on In_*x*_Al_1−*x*_N, where less consensus was reached in the literature previously. Considering the available InAlN data, including those recently reported for low indium contents, empirical formulae for InAlN bandgap and bandgap bowing parameter are proposed. Applying the generalised bandgap data, the refractive index dispersion data available in the literature for III-N alloys is fitted using the Adachi model. For this purpose, a formalism involving a parabolic dependence of the Adachi parameters on the dimensionless bandgap ${\xi }_{{E}_{\mathrm{g}}}=\left({E}_{\mathrm{g}, {\mathrm{A}}_{x}{\mathrm{B}}_{1-x}\mathrm{N}}-{E}_{\mathrm{g},\mathrm{ BN}}\right)/\left({E}_{\mathrm{g}, \mathrm{AN}}-{E}_{\mathrm{g},\mathrm{ BN}}\right)$ of the corresponding ternary alloys is used rather than one directly invoking the alloy composition.

## Introduction

Wurtzite group III-nitrides, including binaries (GaN, InN, and AlN), ternary (In_*x*_Ga_1−*x*_N, Al_*y*_Ga_1−*y*_N and In_*z*_Al_1−*z*_N) and quaternary (In_*p*_Al_*q*_Ga_1−*p*−*q*_N) alloys, are considered to be one of the most important semiconductor materials of today. The direct bandgap across all compositions and the wide bandgap range achievable with III-nitrides makes them interesting for a variety of photonic, electronic and optoelectronic device applications particularly including laser diodes (LDs)^1^, light emitting diodes (LEDs)^2^, high-power and high-frequency amplifiers^3^, photodetectors^4^, etc. Knowledge of the band structure and availability of reliable data on the optical properties of all III-nitride semiconductors are essential to utilise their full application potential. Of these, InAlN is one of the most challenging among III-N ternary alloys to prepare and is thus less studied, with its data being still sparse in the literature.

Here we review and summarise the bandgap and bandgap bowing parameter of the In_*x*_Al_1−*x*_N alloy for the whole composition range and introduce an empirical formula for the InAlN bandgap and its bowing parameter. These are partly based on the data previously available in the literature for indium contents around, and also higher than that corresponding to, InAlN lattice-matched to GaN. These data are combined with those for low indium content (*x* < 0.11) that have relatively recently become available^5^. Finally, the InAlN bandgap data reported here, and such data for AlGaN and InGaN from the literature are used to make estimates of refractive index dispersion for III-N ternary alloys using the Adachi model^6^. These data are of wide application to device modelling and design through control of carrier injection and light extraction, optimisation of active region carrier confinement, laser waveguide^7^ and Bragg reflectors^8,9^.

## Results and discussion

### Bandgap

The simplest possible prediction of a physical property of a ternary semiconductor alloy is to assume it changes linearly between the values at its endpoints corresponding to the relevant binary compounds. This is generally considered to be valid in the case of lattice constant variation with composition and is expressed using Vegard’s law^10^. It has been applied to many semiconductor materials to measure alloy composition, using X-ray diffraction data, for example. However, for most semiconductor materials, the bandgap of an alloy ${E}_{g}^{{\mathrm{A}}_{x}{\mathrm{B}}_{1-x}}$ does not follow a linear Vegard’s law with composition; and some degree of deviation is typically observed. This is commonly accounted for through the inclusion of a so-called “bowing parameter” *b*, leading to a parabolic dependence^11^:

1 $${E}_{g}^{{\mathrm{A}}_{x}{\mathrm{B}}_{1-x}}\left(x\right)={x E}_{g}^{\mathrm{A}}+\left(1-\mathrm{x}\right) {E}_{g}^{\mathrm{B}}-b x \left(1-x\right).$$

Here ${E}_{g}^{\mathrm{A}}$ and ${E}_{g}^{\mathrm{B}}$ are the bandgaps of the compounds A and B, and A_*x*_B_1–*x*_ is the alloy with the relative molar fractions defined by *x*. Generally, an accurate determination of the bowing parameter, *b,* requires knowledge of sample quality, precise composition and strain condition^12,13^. Variation in these values is what leads to the diversity in the reported values of bowing parameters for all III-nitride ternary alloys for instance.

For most common semiconductor alloys, *b* is a relatively small and constant parameter (composition-independent)^11,14,15^. A good example of such an alloy is AlGaN. Although particular values of its bandgap bowing parameter slightly vary (0.86–1.00 eV) between different studies^16–20^ most probably due to the above-mentioned factors, the AlGaN bandgap can be satisfactorily described with a constant (composition independent) bowing parameter of 0.94 eV^21^. In-containing ternary III-N alloys, however, behave differently. For InGaN, for instance, single *b* values have been reported, but the value scatter is large (1.4–3 eV) and the results tend to be proposed based on limited compositional ranges^12,22–27^. Less success in obtaining a good fit has been evaluated when the full compositional range was analysed^13,19,28^. The reason for this is that, *b*, for InGaN, shows a fairly small but still determinable composition-dependence, which is also observed in theoretical reports^29–31^.

In the case of InAlN alloys, there is a particularly large range of reported bowing parameter values inferred from bandgap data^7,29,32–43^. As for InGaN, these reported values tend to be associated with data over a limited composition range. The earliest estimation of *b* was compromised also by the use of the wrong value of 1.9 eV for the InN bandgap^44^. This for a long while was overestimated due to a strong blueshift of the apparent bandgap due to the Moss–Burstein effect in early poor-quality InN layers. These samples were heavily unintentionally doped with oxygen so that the high equilibrium electron concentration made them strongly degenerate^45^. The issue of unintentional Ga auto-incorporation into InAlN discovered recently^46,47^, also provides additional uncertainty to the bandgap and bandgap bowing parameter, as does the reported composition immiscibility between InN and AlN^48^.

It can be seen from the reported experimental data and theoretical calculations that the bandgap bowing parameter tends to change from about 2.5 eV for InAlN with high In content to estimates assumed to be above 10 eV for low indium content. Therefore, the concept of a composition-dependent *b* has been suggested by several groups^15,29,43,49^, who proposed various, in essence empirical, non-parabolic models for the composition dependence of InAlN bandgap.

As early as in 2001, Vurgaftman et al.^15^ suggested a composition-dependent value of *b*, described by the equation of *b*(*x*) = 16 – 9.1 *x*. However, the overestimated value of 1.9 eV was still used in this review paper for the indium nitride bandgap ${E}_{g}^{InN}$. In 2008, Iliopoulos et al.^50^ suggested another empirical formula for the composition-dependent bowing parameter of InAlN to describe their experimental data:

2 $${b}^{{In}_{x}{Al}_{1-x}N}\left(x\right)=\frac{15.3}{1+4.8 x}$$

Later, Sakalauskas et al.^49^ modified the Iliopoulos et al. formula and proposed the new empirical expression for the composition-dependent InAlN bandgap bowing parameter:

3 $${b}^{{In}_{x}{Al}_{1-x}N}\left(x\right)=\frac{A}{1+C {x}^{2}}$$

where *A* and *C* are fitting parameters, found to be 6.43 ± 0.12 eV and 1.21 ± 0.14 eV, respectively.

**Figure 1:**
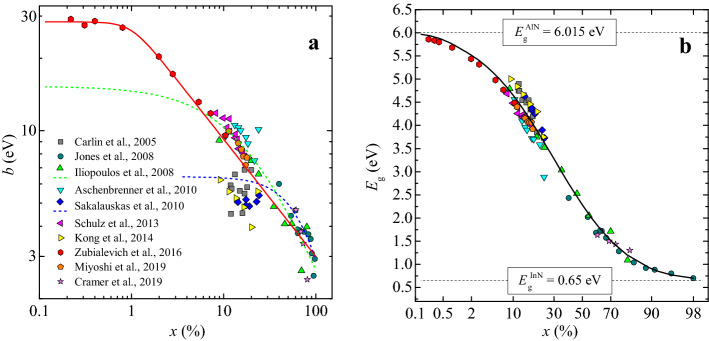
InAlN bandgap bowing parameter (**a**) and band-gap (**b**) with their best fit using (4) for *b*(*x*) and (1) for *E*_*g*_(*x*) across the full range of In contents, in combination with other previously reported data from Refs.^5,7,42,43,49–55^.

The main drawback of the above-mentioned studies was the absence of reliable experimental data for low indium content InAlN bandgaps so that the behaviour for $x<0.1$ is simply extrapolated from trends in the rest of the composition range. This explains particularly the large discrepancy between these extrapolated bowing parameters (Fig. 1a) and opens questions on the reliability of such estimations in the low indium content range.

A further challenge in determining a reliable composition-dependent bandgap is that most reported data is self-consistent within its own set, and hard to compare with other sets, making any generalisation from the available data non-trivial. This is particularly seen in the large scatter in bandgap data around the most studied In_*x*_Al_1−*x*_N compositions, which are those close to being lattice-matched to GaN (at *x* ≈ 0.18), as seen in Fig. 1b.

Our group has recently reported an accurate data set for *x* < 0.11^5^. Based on this review and analysis of all known previous reported data, a revised empirical model for the composition-dependent bowing parameter of the In_*x*_Al_1–*x*_N bandgap across the entire alloy range can be proposed, giving the experimental fit presented in Fig. 1a, where the value of *b*(*x*) is given by:

4 $$b\left(x\right)=\frac{{b}_{0}}{{\left(1+{(x/{x}_{0})}^{n}\right)}^{s}} .$$

Here *b*_0_ is the bowing parameter in the *x* → 0 limit; *x*_0_ is the indium content where the bowing parameter remains almost constant below it (*b*(*x*) ≈ *b*_0_), *n* and *s* are additional parameters defining the ultimate slope of log(*b*) and showing how quick this slope is reached at *x ≥ x*_0_. As the *n* and *s* parameters can partially compensate each other leading to a range of potential good fits, we set *n* = 4, with the rest of the empirical parameters that fitted as following: *b*_0_ = 28.3 ± 0.9 eV, *x*_0_ = 0.0100 ± 0.0017 and *s* = 0.122 ± 0.007. These parameters differ slightly from the best fit of the bowing parameter restricted to the low *x* region only^5^.

InAlN bandgaps in the full composition range calculated with Eq. (1), using the above-described bowing parameter and bandgaps of AlN and InN binaries (${E}_{\mathrm{g}}^{\mathrm{AlN}}$ = 6.015 eV^56^ and ${E}_{\mathrm{g}}^{\mathrm{InN}}$ = 0.65 eV^57–59^), are presented in Fig. 1b, showing a good fit across the full range of experimental data reported in the literature.

**Figure 2:**
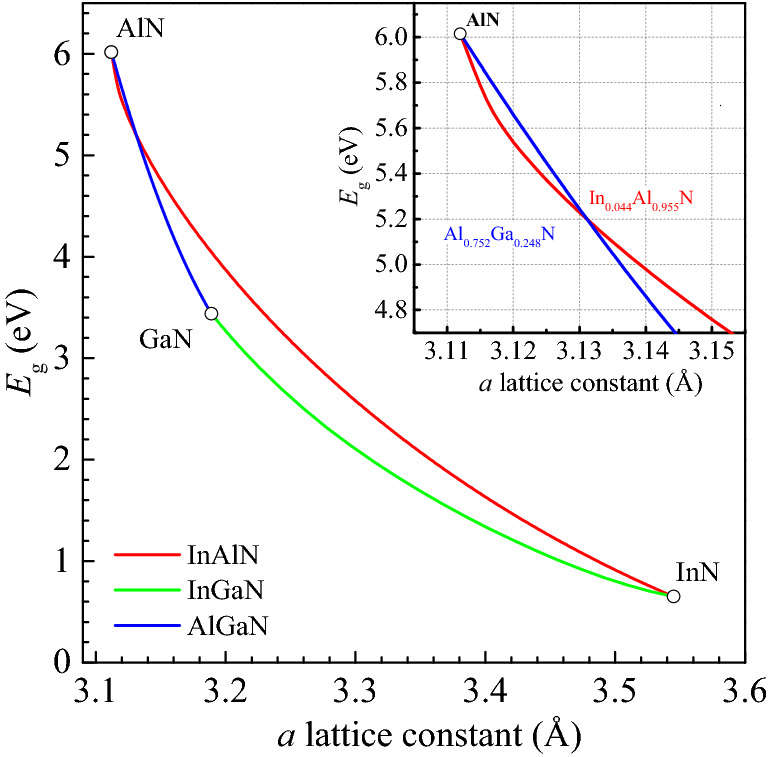
Bandgap–*a* lattice parameter diagram for ternary III-nitride alloys. Bandgap data: InAlN – this work, AlGaN^21^, InGaN^31^.

Using the data for InAlN presented above, we can now provide an updated bandgap–lattice parameter diagram for all III-nitrides at room temperature. For this purpose, we use bandgap endpoints of 0.65, 3.438 and 6.015 eV for InN^57–59^, GaN^60^ and AlN^56^, respectively. We have considered using the same empirical model (Eq. (4)) for the InGaN and AlGaN systems, but their deviations from the constant (with composition) bowing parameter are regarded as too small to justify this approach, taking into account the spread and uncertainties in the experimental data available. Therefore, bandgap bowing parameters for InGaN and AlGaN from Refs 21,31 that match pretty well the majority of previously published data have been applied instead. Vegard’s law is used to interpolate between the *a* lattice parameters of InN (3.545 Å), GaN (3.189 Å), and AlN (3.112 Å)^61^ for the ternary alloys. The resulting bandgap–lattice parameter diagram is depicted in Fig. 2. It is seen from the diagram that there is a narrow range close to pure AlN where the InAlN bandgap is actually lower than that of lattice-matched AlGaN with a specific point (In_0.044_Al_0.956_ N, Al_0.751_Ga_0.249_ N) where both the bandgap and the lattice parameter of the two alloys are equal (approx. 5.2 eV and 3.131 Å, respectively). The existence of such a range is due to the very high bandgap bowing parameter of InAlN as one approaches the low indium composition limit, as described above.

It is worth noting that the largest bandgap contrast (> 0.5 eV) for InAlN lattice-matched to AlGaN or InGaN occurs for lattice parameters between 3.180 Å and 3.235 Å (i.e. approximately between Al_0.01_Ga_0.99_ N and In_0.2_Ga_0.8_ N). The rapid decrease in bandgap difference for lattice parameters less than 3.18 Å and the similarity of bandgaps of the lattice-matched alloys for *E*_g_ > 5 eV will present potential challenges in using InAlN for deep UV (< 300 nm) optoelectronics.

### Refractive index

In addition to the band-structure properties of III-nitride binary and ternary alloys, dispersion of their refractive index is another key point of interest. There are a few techniques suggested in the literature to determine the refractive index of alloy semiconductors. Using an empirical oscillator model is very common to determine the dispersion of the refractive index, for energies lower than the band-gap energy^62^. Particularly, the Sellmeier empirical expression is widely used when fitting experimental data for the refractive index dispersion of dielectric materials^63^:

5 $$n\left(\lambda \right)=\sqrt{A+\frac{{\lambda }^{2}}{{\lambda }^{2}-B}} ,$$

where λ is the light wavelength, and *A* and *B* are parameters determined by fitting to experimental data. To find these coefficients for alloy semiconductors, Sellmeier parameters are typically interpolated. Particularly, Takeuchi et al.^63^ suggested the following composition-dependent parameters *A*(*x*) and *B*(*x*) for Al_*x*_Ga_1−*x*_N alloys:

6 $$ \begin{aligned} A\left( x \right) & = 4.27 - 1.07 x, \\ B\left( x \right) & = 0.092 - 0.065 x. \\ \end{aligned} $$

The parameters *A*(*x*) and *B*(*x*) were obtained by fitting experimental values across the composition range and managed to deal effectively with AlGaN alloys^63,64^. However, based on our knowledge, there are currently no such systematic values for InAlN and InGaN materials.

Although the Sellmeier equation is valid in such cases, the refractive-index dispersion obtained from this expression is not directly based on the Kramers–Kronig relations. Furthermore, due to its semi-empirical nature, the modified Sellmeier equation, particularly the first-order one as in Eq. (5), has only a limited capability in describing experimental dispersions of refractive indices of transparent materials. For this reason, in the case of semiconductors, it can be valid across only limited spectral ranges, with better results well below bandgap^65^.

A more physics-based model to describe the dielectric function of dielectric materials was proposed by Adachi in 1982^6^. This model is applicable also to semiconductors in their transparency spectral range, i.e. for photon energies lower than the bandgap ($E<{E}_{g}$)^66^:

7 $$n\left(E\right)=\sqrt{a{\left(\frac{E}{{E}_{g}}\right)}^{-2}\left[2-{\left(1+\left(\frac{E}{{E}_{g}}\right)\right)}^\frac{1}{2}-{\left(1-\left(\frac{E}{{E}_{g}}\right)\right)}^\frac{1}{2}\right]+b} ,$$

where *n*(*E*) is the dependence of refractive index on photon energy, and *a* and *b* are fitting parameters, which for alloys are composition (and thus bandgap) dependent. This formula has been widely and successfully used to fit experimental data for the III-nitride binary materials (AlN, GaN and InN)^66,67^. A linear interpolation of the parameters *a* and *b* used for binary materials can also be used to determine the refractive index of the ternary alloys as reported by^66–68^.

Peng and Piprek obtained the parameters *a* and *b* by linear interpolation from the binary III-nitrides for ternary compounds in 1996^35,66^. The parameters proposed by Peng and Piprek for wurtzite GaN, AlN and InN are summarised in Table 1.

**Table 1:** Parameters of wurtzite GaN, AlN and InN used by Peng and Piprek ^66^.

Material	*E*_g_ (eV)	*a*	*b*
InN	1.9	53.57	− 9.19
GaN	3.4	9.31	3.03
AlN	6.2	13.55	2.05

Subsequently, in 1997, Piprek et al.^69^ proposed a non-linear interpolation for Al_*x*_In_1−*x*_N alloys. Although the parameters and formulas proposed by Peng and Piprek worked to some level of accuracy, they were still far from ideal values to use in advanced and sensitive applications like laser waveguide design. The major issues in the results reported in Ref.^69^ include the use of 1.9 eV for InN bandgap^70^. This, in particular, explains the significant difference in the parameters *a* and *b* for InN in comparison to those for AlN and GaN (Table 1).

To get a better level of accuracy, Laws et al.^67^ introduced their modified non-linear expressions for the parameters *a*(*x*) and *b*(*x*). For Al_*x*_Ga_1−*x*_N in the range of 0 < *x* < 0.38 the formulae would be as in Eqs. (8):

8 $$ \begin{aligned} a\left( x \right) & = 9.827 {-} 8.216 x {-} 31.59 x^{2} , \\ b\left( x \right) & = 2.736 + 0.842 x {-} 6.29 x^{2} . \\ \end{aligned} $$

As these parameters still do not cover the full composition range, a further degree of generalisation is required to describe available experimental data.

To determine the dispersion of refractive index of ternary nitrides here, we are also applying the Adachi model using the binary and ternary bandgap values discussed earlier (see Fig. 2, for example). As *c*-plane oriented material is most commonly used in various applications, the fundamental bandgap of AlN (6.015 eV) from the view of refractive index is to be substituted with its apparent bandgap of 6.24 eV (${E}_{\mathrm{g}}^{\mathrm{AlN}}$ + Δ_CF_ = 6.015 + 0.225 eV)^56,71^, where Δ_CF_ is VB crystal-field splitting. It separates the uppermost valence subband of *c*-plane AlN, transitions from which to its CB are forbidden when excited by light with ***E*** $\perp$*c* (normal incidence of light), from those for which such transitions are allowed.

Using previously published experimental data for ordinary refractive indexes of AlN^49,68,72–74^, GaN^18,72,74–77^ and InN^76,77^ enabled us to obtain state-of-the-art *a* and *b* parameters of these binary nitrides. Energy bandgap and Adachi fitting parameters of wurtzite AlN, GaN and InN are summarised in Table 2.

**Table 2:** Bandgap and Adachi model fitting parameters of wurtzite InN, GaN and AlN.

Material	*E*_*g*_ (eV)	*a*	*b*
InN	0.65	9.4 ± 0.5	5.0 ± 0.2
GaN	3.438	10.1 ± 0.3	2.67 ± 0.08
AlN	6.24^a^	17.6 ± 0.9	− 0.3 ± 0.2

^a^Apparent bandgap as explained in the text.

The most straightforward approach to obtain the refractive index dispersion for ternary alloys would be to use their known bandgaps and a linear interpolation of *a* and *b* parameters between the corresponding binaries’ endpoints. However, as was pointed out by Laws et al.^67^, even for AlGaN the best results can be achieved when the fitting parameters have a nonlinear dependence on the alloy composition. This issue is anticipated to be even more critical in In-containing ternary III-Ns. Thus, a more feasible approach would involve a direct analysis of available experimental data on refractive index dispersion of the ternary alloys as described below.

The second point worth noting is that due to the discrepancies in reported bandgaps as a function of composition, the fitting parameters for the refractive index are to be applied as a function of bandgap. This has been noted and successfully applied by Özgür et al.^72^ with regard to the parameters of Sellmeier relation (5) for AlGaN refractive index dispersion. For InAlN, it is especially important, as the discrepancies are particularly large for this alloy (Fig. 1b) and as the refractive index is indeed a function of alloy bandgap and not directly of its composition. For convenience, however, we found it suitable to use dimensionless bandgap parameters^24^ (${\xi }_{{E}_{g}}$) as arguments for *a* and *b* parameters:

9 $${\xi }_{{E}_{g}}=\frac{{E}_{\mathrm{g}}^{{\mathrm{A}}_{x}{\mathrm{B}}_{1-x}}-{E}_{\mathrm{g}}^{\mathrm{B}}}{{E}_{\mathrm{g}}^{\mathrm{A}}-{E}_{\mathrm{g}}^{\mathrm{B}}},$$

where A, B and A_*x*_B_1–*x*_ are two binary III-Ns and their alloy, respectively.

**Figure 3:**
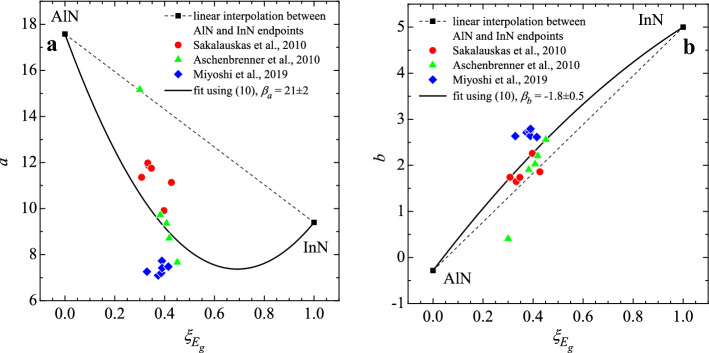
Adachi (**a**) *a* and (**b**) *b* parameters for AlN and InN endpoints (black squares) taken from Table 2 and ternary InAlN alloys (coloured figures) derived from^7,49,51,52^ as a function of dimensionless bandgap ${\xi }_{{E}_{g}}$. Thin dashed lines show linear interpolations between the endpoints, and the solid lines correspond to fitting with Eq. (10). (It should be noted that *b* here is different from the bandgap bowing parameter “*b*” represented earlier).

We considered (the rather scarce) available experimental data on InAlN alloy refractive index dispersion^7,49,51,52^, by fitting them with Eq. (7) and calculating corresponding dimensionless bandgaps ${\xi }_{{E}_{g}}$. The obtained results are presented in Fig. 3. Although the available literature data is very limited and rather severely scattered, it is clearly visible that there is no linear dependency of either parameter on the dimensionless bandgap. For better fit thus a bowing is introduced, and we propose it in the same way as in Eq. (1):

10 $${p}_{i}^{\mathrm{InAlN}}={p}_{i}^{\mathrm{InN}}\cdot {\xi }_{{E}_{\mathrm{g}}}+{p}_{i}^{\mathrm{AlN}}\cdot \left(1-{\xi }_{{E}_{\mathrm{g}}}\right)-{\beta }_{{p}_{i}}\cdot {\xi }_{{E}_{\mathrm{g}}}\cdot \left(1-{\xi }_{{E}_{\mathrm{g}}}\right), i \in 1, 2$$

where parameters *p*_1_, *p*_2_ are *a* and *b*, respectively, and thus *β*_*a*_, *β*_*b*_ are corresponding bowing parameters.

**Figure 4:**
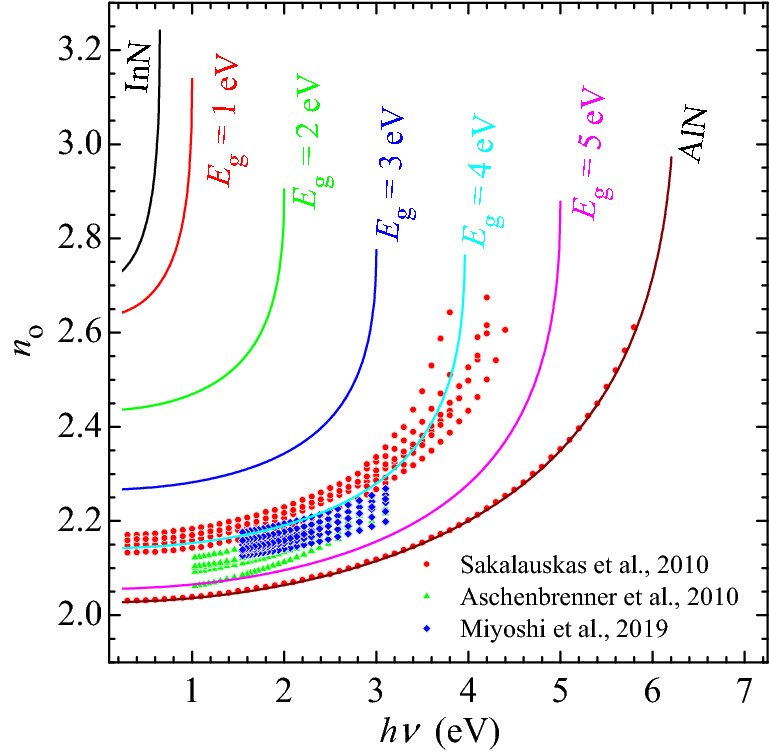
Dispersion of refractive index of different bandgap InAlN alloys: dots—experiment from^7,49,51,52^, lines—modelled with Adachi approach (7) where the *a* and *b* parameters are calculated using (10) with endpoints from Table 2 and corresponding bowing parameters from Fig. 3.

Figure 4 shows the result of refractive index dispersion modelling using this approach for InAlN with bandgaps between 1.0 eV and 5.0 eV, with a 1.0 eV step. Taking into account the large scattering in available data on the refractive index, which can exceed 0.1 for InAlN with similar bandgap reported by different authors, there is a limit to the quality of fitting that can be achieved. The discrepancy between the proposed formula and an arbitrary reported experimental refractive index dispersion datum can be as high as 0.07, and thus needs to be used with caution. Nevertheless, we believe it is the best estimation could be made using the InAlN data available to date.

We followed the same methodology in analysing experimental data for AlGaN^18,72,73^ and InGaN^77,78^, and the resulting bowing parameters *β*_*a*_ and *β*_*b*_ are given in Table 3. It is worth noting the relatively small bowing found in the case of InGaN. We consider this to be due to the particularly strong scattering of available data, even within the same InGaN sample series. This makes the uncertainties in the bowing parameter values very high. Taking this into consideration, it may be appropriate not to apply a bowing function for the Adachi parameters for InGaN, until more data for this alloy becomes available.

**Table 3:** Extracted bowing parameters for Adachi model parameters *a* and *b* for ternary III-Ns.

Alloy	*β*_*a*_	*β*_*b*_
InAlN	21 ± 2	− 1.8 ± 0.2
AlGaN	4.3 ± 1.7	− 0.7 ± 0.5
InGaN	3 ± 4	1.4 ± 1.1

We propose the following procedure to obtain an appropriate best estimate of the III-N refractive index for model purposes. Starting at the desired alloy composition, first calculate the bandgap (for InAlN, using Eqs. (1) and (4) as appropriate). Next, calculate the corresponding dimensionless bandgap using Eq. (9), which in turn allows the estimation of $a({\xi }_{{E}_{g}})$ and $b({\xi }_{{E}_{g}})$ from (10), where bowing parameters *β*_*a*_ and *β*_*b*_ are from Table 3. Finally, the Adachi expression (7) can be used for calculation of the refractive index dispersion. Accordingly, if an experimental measurement can provide an independent bandgap data then this option will be preferable to inferring a bandgap from a composition.

Due to the very limited and/or scattered experimental data on the refractive index of InAlN and InGaN, the values here represent the best estimate to the problem rather than a definitive solution at this stage. Another concern in using this Adachi approach, which perhaps in particular important for In containing alloys, is that even at moderate indium contents the absorption edge is broadened. This can lead to the refractive index dispersion becoming convex in the spectral region near bandgap^79^ rather than being concave, which the Adachi formula would predict. This issue is anticipated to be less prominent in multiple QW stacks, short-period superlattices, and in general for layers of low indium content, than in thick epitaxial layers of moderate-to-high indium contents. It would require substantial further analysis to incorporate this behaviour into a predictive model.

## Summary and conclusions

Using reported bandgap data for In_*x*_Al_1–*x*_N with indium content above 0.1 and our recent accurate data for *x* ≤ 0.11, empirical expressions for the bandgap and the bandgap bowing parameter of InAlN alloys across the full composition range are proposed. Together with best, in our opinion, literature data on AlGaN and InGaN, this new InAlN dataset allowed to update the bandgap-lattice parameter diagram for III-nitrides.

Previous particular attempts of using Adachi approach for estimation of III-N refractive index dispersion have been updated and generalised to all III-nitrides and their ternary alloys. For this, the best estimate bandgaps of the compound alloys are considered including particularly those for InAlN reported here. It was determined from the direct analysis of previously published experimental data on refractive index dispersion of III-N ternary alloys that the fitting parameters of the Adachi expression cannot be interpolated linearly between those for corresponding binaries and that bowing is to be introduced. The bowing was found to be significantly higher for InAlN in comparison to AlGaN, while only a limited estimation was possible for InGaN due to the extremely scattered literature data currently available.

In conclusion, the data on bandgap, bandgap bowing and refractive index dispersion for InAlN and other ternary III-Ns are reported, revised and suggestions made for the most appropriate current values to apply in the design and optimisation of III-nitride-based optical, photonic and optoelectronic devices and structures.

## References

[1] Nakamura S, Pearton S, Fasol G (2013). The Blue Laser Diode: The Complete Story.

[2] Seong T-Y, Han J, Amano H, Morkoç H (2013). III-Nitride Based Light Emitting Diodes and Applications.

[3] Mishra UK, Parikh P, Wu Y-F (2002). AlGaN/GaN HEMTs—an overview of device operation and applications. Proc. IEEE.

[4] Munoz E (2001). III nitrides and UV detection. J. Phys. Condens. Matter.

[5] Zubialevich VZ (2016). Strongly nonparabolic variation of the band gap in In_*x*_Al_1−__*x*_N with low indium content. Semicond. Sci. Technol..

[6] Adachi S (1982). Refractive indices of III–V compounds: key properties of InGaAsP relevant to device design. J. Appl. Phys..

[7] Aschenbrenner T (2010). Optical and structural characterization of AlInN layers for optoelectronic applications. J. Appl. Phys..

[8] Carlin J-F, Ilegems M (2003). High-quality AlInN for high index contrast Bragg mirrors lattice matched to GaN. Appl. Phys. Lett..

[9] Zhang L (2013). Solar-blind ultraviolet AlInN/AlGaN distributed Bragg reflectors. Appl. Phys. Lett..

[10] Vegard L (1921). Die konstitution der mischkristalle und die raumfüllung der atome. Z. Phys..

[11] Van Vechten JA, Bergstresser TK (1970). Electronic structures of semiconductor alloys. Phys. Rev. B.

[12] Yan Q, Rinke P, Janotti A, Scheffler M, Van de Walle CG (2014). Effects of strain on the band structure of group-III nitrides. Phys. Rev. B.

[13] Orsal G (2014). Bandgap energy bowing parameter of strained and relaxed InGaN layers. Opt. Mater. Express.

[14] Ebina A, Fukunaga E, Takahashi T (1974). Variation with composition of the *E*_0_ and *E*_0_+Δ_0_ gaps in ZnS_*x*_Se_1−__*x*_ alloys. Phys. Rev. B.

[15] Vurgaftman I, Meyer JR, Ram-Mohan LR (2001). Band parameters for III–V compound semiconductors and their alloys. J. Appl. Phys..

[16] Teofilov N (2002). Optical investigation of Al_*x*_Ga_1−__*x*_N epitaxial films grown on AlN buffer layers. Diam. Relat. Mater..

[17] Nam KB, Li J, Nakarmi ML, Lin JY, Jiang HX (2004). Unique optical properties of AlGaN alloys and related ultraviolet emitters. Appl. Phys. Lett..

[18] Buchheim C (2005). Dielectric function and critical points of the band structure for AlGaN alloys. Phys. Status Solidi B.

[19] Pelá RR (2011). Accurate band gaps of AlGaN, InGaN, and AlInN alloys calculations based on LDA-1/2 approach. Appl. Phys. Lett..

[20] Jmerik VN, Lutsenko EV, Ivanov SV (2013). Plasma-assisted molecular beam epitaxy of AlGaN heterostructures for deep-ultraviolet optically pumped lasers. Phys. Status Solidi A.

[21] Coughlan C, Schulz S, Caro MA, O'Reilly EP (2015). Band gap bowing and optical polarization switching in Al_1−__*x*_Ga_*x*_N alloys. Phys. Status Solidi B.

[22] Kuo Y-K, Lin W-W, Lin J (2001). Band-gap bowing parameter of the In_*x*_Ga_1–__*x*_N derived from theoretical simulation. Jpn. J. Appl. Phys..

[23] Wu J (2002). Small band gap bowing in In_1−__*x*_Ga_*x*_N alloys. Appl. Phys. Lett..

[24] Wu J (2003). Universal bandgap bowing in group-III nitride alloys. Solid State Commun..

[25] Kurouchi M (2004). Growth and properties of In-rich InGaN films grown on (0001) sapphire by RF-MBE. Phys. Status Solidi B.

[26] Moret M (2009). Optical, structural investigations and band-gap bowing parameter of GaInN alloys. J. Cryst. Growth.

[27] Islam MR, Kaysir MR, Islam MJ, Hashimoto A, Yamamoto A (2013). MOVPE growth of In_x_Ga_1−x_N (x ∼ 0.4) and fabrication of homo-junction solar cells. J. Mater. Sci. Technol..

[28] Wang K (2008). Optical energies of AlInN epilayers. J. Appl. Phys..

[29] Ferhat M, Bechstedt F (2002). First-principles calculations of gap bowing in In_*x*_Ga_1−__*x*_N and In_*x*_Al_1−__*x*_N alloys: relation to structural and thermodynamic properties. Phys. Rev. B.

[30] Moses PG, Walle CGVD (2010). Band bowing and band alignment in InGaN alloys. Appl. Phys. Lett..

[31] Caro MA, Schulz S, O’Reilly EP (2013). Theory of local electric polarization and its relation to internal strain: Impact on polarization potential and electronic properties of group-III nitrides. Phys. Rev. B.

[32] Kubota K, Kobayashi Y, Fujimoto K (1989). Preparation and properties of III-V nitride thin films. J. Appl. Phys..

[33] Guo Q, Ogawa H, Yoshida A (1995). Growth of Al_*x*_In_1−__*x*_N single crystal films by microwave-excited metalorganic vapor phase epitaxy. J. Cryst. Growth.

[34] Kim KS, Saxler A, Kung P, Razeghi M, Lim KY (1997). Determination of the band-gap energy of Al_1−__*x*_In_*x*_N grown by metal–organic chemical-vapor deposition. Appl. Phys. Lett..

[35] Peng T (1997). Band gap bowing and refractive index spectra of polycrystalline Al_*x*_In_1−__*x*_N films deposited by sputtering. Appl. Phys. Lett..

[36] Yamaguchi S (1998). Structural and optical properties of AlInN and AlGaInN on GaN grown by metalorganic vapor phase epitaxy. J. Cryst. Growth.

[37] Lukitsch MJ (2001). Optical and electrical properties of Al_1−__*x*_In_*x*_N films grown by plasma source molecular-beam epitaxy. Appl. Phys. Lett..

[38] Dridi Z, Bouhafs B, Ruterana P (2003). First-principles investigation of lattice constants and bowing parameters in wurtzite Al_*x*_Ga_1–__*x*_N, In_*x*_Ga_1–__*x*_N and In_*x*_Al_1–__*x*_N alloys. Semicond. Sci. Technol..

[39] Onuma T (2003). Recombination dynamics of localized excitons in Al_1−__*x*_In_*x*_N epitaxial films on GaN templates grown by metalorganic vapor phase epitaxy. J. Appl. Phys..

[40] Vurgaftman I, Meyer JR (2003). Band parameters for nitrogen-containing semiconductors. J. Appl. Phys..

[41] Butté R (2005). Recent progress in the growth of highly reflective nitride-based distributed bragg reflectors and their use in microcavities. Jpn. J. Appl. Phys..

[42] Jones RE (2008). Band gap bowing parameter of In_1−__*x*_Al_*x*_N. J. Appl. Phys..

[43] Schulz S (2013). Composition-dependent band gap and band-edge bowing in AlInN: a combined theoretical and experimental study. Appl. Phys. Express.

[44] Davydov VY (2002). Absorption and emission of hexagonal InN evidence of narrow fundamental band gap. Phys. Status.

[45] Wu J (2004). Effects of electron concentration on the optical absorption edge of InN. Appl. Phys. Lett..

[46] Taylor E (2014). Structural and optical properties of Ga auto-incorporated InAlN epilayers. J. Cryst. Growth.

[47] Smith MD (2014). Determination of Ga auto-incorporation in nominal InAlN epilayers grown by MOCVD. J. Mater. Chem. C.

[48] Matsuoka T (1997). Calculation of unstable mixing region in wurtzite In_1−__*x*__−__*y*_Ga_*x*_Al_*y*_N. Appl. Phys. Lett..

[49] Sakalauskas E (2010). Dielectric function and optical properties of Al-rich AlInN alloys pseudomorphically grown on GaN. J. Phys. D Appl. Phys..

[50] Iliopoulos E, Adikimenakis A, Giesen C, Heuken M, Georgakilas A (2008). Energy bandgap bowing of InAlN alloys studied by spectroscopic ellipsometry. Appl. Phys. Lett..

[51] Miyoshi M, Yamanaka M, Egawa T, Takeuchi T (2019). Microstructure variation in thick AlInN films grown on *c*-plane GaN on sapphire by metalorganic chemical vapor deposition. J. Cryst. Growth.

[52] Miyoshi M, Yamanaka M, Egawa T, Takeuchi T (2019). A 300 nm thick epitaxial AlInN film with a highly flat surface grown almost perfectly lattice-matched to *c*-plane free-standing GaN substrate. Jpn. J. Appl. Phys..

[53] Carlin J-F (2005). Progresses in III-nitride distributed Bragg reflectors and microcavities using AlInN/GaN materials. Phys. Status Solidi B.

[54] Kong W (2014). Room temperature photoluminescence from In_*x*_Al_(1–__*x*__)_N films deposited by plasma-assisted molecular beam epitaxy. Appl. Phys. Lett..

[55] Cramer RC, Kyle ECH, Speck JS (2019). Band gap bowing for high In content InAlN films. J. Appl. Phys..

[56] Feneberg M, Leute RAR, Neuschl B, Thonke K, Bickermann M (2010). High-excitation and high-resolution photoluminescence spectra of bulk AlN. Phys. Rev. B.

[57] Fu S, Chen T, Chen Y (2006). Photoluminescent properties of InN epifilms. Semicond. Sci. Technol..

[58] Nanishi Y, Saito Y, Yamaguchi T (2003). RF-molecular beam epitaxy growth and properties of InN and related alloys. Jpn. J. Appl. Phys..

[59] Wu J (2003). Temperature dependence of the fundamental band gap of InN. J. Appl. Phys..

[60] Piprek, J. & Li, S. in *Optoelectronic Devices: Advanced Simulation and Analysis* (ed Joachim Piprek) 293–312 (Springer New York, 2005). 10.1007/0-387-27256-9_10

[61] Morkoç H (2009). Handbook of Nitride Semiconductors and Devices.

[62] Piprek J (2013). Semiconductor Optoelectronic Devices: Introduction to Physics and Simulation.

[63] Takeuchi K, Adachi S, Ohtsuka K (2010). Optical properties of Al_*x*_Ga_1−__*x*_N alloy. J. Appl. Phys..

[64] Touré A (2012). Characterization of low Al content Al_*x*_Ga_1−__*x*_N epitaxial films grown by atmospheric-pressure MOVPE. Phys. Status Solidi A.

[65] Adachi S (1999). Optical Properties of Crystalline and Amorphous Semiconductors: Materials and Fundamental Principles.

[66] Peng T, Piprek J (1996). Refractive index of AlGaInN alloys. Electron. Lett..

[67] Laws G, Larkins E, Harrison I, Molloy C, Somerford D (2001). Improved refractive index formulas for the Al_*x*_Ga_1−__*x*_N and In_*y*_Ga_1−__*y*_N alloys. J. Appl. Phys..

[68] Brunner D (1997). Optical constants of epitaxial AlGaN films and their temperature dependence. J. Appl. Phys..

[69] Piprek, J., Peng, T., Qui, G. & Olowolafe, J. in *1997 IEEE International Symposium on Compound Semiconductors.*227–230 (IEEE).

[70] Wu J, Walukiewicz W (2003). Band gaps of InN and group III nitride alloys. Superlattices Microstruct..

[71] Feneberg M (2011). Synchrotron-based photoluminescence excitation spectroscopy applied to investigate the valence band splittings in AlN and Al_0.94_Ga_0.06_N. Appl. Phys. Lett..

[72] Özgür Ü, Webb-Wood G, Everitt HO, Yun F, Morkoç H (2001). Systematic measurement of Al_*x*_Ga_1−__*x*_N refractive indices. Appl. Phys. Lett..

[73] Antoine-Vincent N (2003). Determination of the refractive indices of AlN, GaN, and Al_*x*_Ga_1−__*x*_N grown on (111) Si substrates. J. Appl. Phys..

[74] Watanabe N, Kimoto T, Suda J (2008). The temperature dependence of the refractive indices of GaN and AlN from room temperature up to 515°C. J. Appl. Phys..

[75] Buchheim C (2009). Influence of anisotropic strain on excitonic transitions in *a*-plane GaN films. Microelectron. J..

[76] Sakalauskas, E. *Optical Properties of Wurtzite InN and Related Alloys* doctor rerum naturalium (Dr. rer. nat.) thesis, Technischen Universität Ilmenau (2012).

[77] Kazazis SA, Papadomanolaki E, Androulidaki M, Kayambaki M, Iliopoulos E (2018). Optical properties of InGaN thin films in the entire composition range. J. Appl. Phys..

[78] Sakalauskas E (2012). Dielectric function and bowing parameters of InGaN alloys. Phys. Status Solidi B.

[79] Gokarna A (2010). Optical and microstructural properties versus indium content in In_*x*_Ga_1−__*x*_N films grown by metal organic chemical vapor deposition. Appl. Phys. Lett..

